# *Notes from the Field*: Differences in Suicide Rates, by Race and Ethnicity and Age Group — United States, 2018–2023

**DOI:** 10.15585/mmwr.mm7435a2

**Published:** 2025-09-18

**Authors:** Deborah M. Stone, Alison L. Cammack, Eric G. Carbone

**Affiliations:** 1 Division of Injury Prevention, National Center for Injury Prevention and Control, CDC.

**Summary**

**What is already known about this topic?**

Suicide is among the top eight leading causes of death among persons aged 10–64 years; rates differed among demographic groups during 2018–2021.

**What is added by this report?**

During 2018–2023, U.S. suicide rates remained stable overall but differed among demographic groups. Rates increased among non-Hispanic Black or African American and Hispanic or Latino persons and decreased among non-Hispanic White persons and persons aged 10–24 years. Rates were highest among non-Hispanic American Indian or Alaska Native persons but declined between 2021 and 2023.

**What are the implications for public health practice?**

Implementation of evidence-based suicide prevention strategies in populations with high or increasing suicide rates could help save lives. CDC’s Suicide Prevention Resource for Action provides a comprehensive approach to prevention, addressing the many factors associated with suicide.

Suicide (death caused by injuring oneself with intent to die) is a serious U.S. public health problem (*1*) and ranks among the top eight leading causes of death among persons aged 10–64 years (FactsAboutSuicideCDC). From 2003 to 2023, age-adjusted suicide rates increased 31% (*2*), reaching 14.2 suicides per 100,000 population in 2018, the highest rate since 1941 (*3*). Suicide rates subsequently declined to 13.9 in 2019 and to 13.5 in 2020 but increased again in 2021 to 14.1 and remained essentially unchanged in 2022 (14.2) and 2023 (14.1) (*2*). This report updates a previous report that noted racial, ethnic, and age group differences in suicide rates during 2018–2021 (*4*) by extending the analytic period through 2023 and examines rate differences between 2021 and 2023. 

## Investigation and Outcomes

### Data Source and Analysis

Data from CDC’s National Vital Statistics System multiple cause-of-death mortality files for 2018–2023 were analyzed (*2*). Age-adjusted suicide rates and 95% CIs were calculated by the directmethod and using the 2000 U.S. standard population with U.S.CensusBureaupostcensalsingle-raceestimates. Hispanic or Latino (Hispanic) ethnicity includes persons of any race, and all named racial groups were considered non-Hispanic. Persons with unknown ethnicity were excluded from race and ethnicity analyses, and, owing to small numbers, children aged <10 years were excluded from age-specific analyses; all suicide deaths were included in the total. Differences in suicide rates from 2018 to 2023 as well as from 2021 to 2023 were compared using z-tests when the number of suicide deaths was at least 100; p-values <0.05 were considered statistically significant. When the number of suicide deaths was less than 100, differences were considered significant if CIs based on a gamma distribution did not overlap. This activity was reviewed by CDC, deemed not research, and conducted consistent with applicable federal law and CDC policy.*

### Overall Age-Adjusted Suicide Rates, by Race and Ethnicity

In 2023, the overall U.S. age-adjusted suicide rate was 14.1 per 100,000. Rates were lowest among non-Hispanic Asian (Asian) persons (6.5) and highest among non-Hispanic American Indian or Alaska Native (AI/AN) (23.8), non-Hispanic White (White) (17.6), and non-Hispanic Native Hawaiian or Pacific Islander (NH/PI) persons (17.3) (Table) (SupplementaryFigure). During 2018–2023, although overall U.S. age-adjusted suicide rates did not change, age-adjusted rates did change by race and ethnicity, increasing significantly among non-Hispanic Black or African American (Black) (25.2%) and Hispanic persons (10.0%) and declining significantly among White persons (3.1%). Rate differences among NH/PI persons were not significant because of small numbers. From 2021 to 2023, overall rates among AI/AN persons declined significantly (15.3%).

**TABLE:** Suicide rates,* rate changes, and annual number of suicide deaths, by race and ethnicity and age group — National Vital Statistics System, United States, 2018–2023

Year, race and ethnicity^†^	Total no. of suicides	Age-adjusted rate^§^	Age group, yrs							
			10–24		25–44		45–64		≥65	
			No. of suicides	Rate	No. of suicides	Rate	No. of suicides	Rate	No. of suicides	Rate
**Total^¶^**										
2018	48,344	14.2	6,807	10.7	15,541	17.9	16,885	20.1	9,102	17.4
2019	47,511	13.9	6,488	10.2	15,584	17.8	16,250	19.5	9,173	17.0
2020	45,979	13.5	6,643	10.5	15,768	17.9	14,409	17.4	9,137	16.4
2021	48,183	14.1	7,126	11.0	16,724	18.8	14,668	17.6	9,652	17.3
2022	49,476	14.2	6,533	10.0	16,848	18.9	15,645	19.0	10,438	18.1
2023	49,316	14.1	6,417	9.9	16,986	18.9	15,469	18.8	10,437	17.6
2023 vs. 2021 relative rate change, %**	NA	0.3	NA	−10.2^††^	NA	0.4	NA	6.9^††^	NA	1.9
2023 vs. 2018 relative rate change, %**	NA	–0.8	NA	−7.0^††^	NA	5.7^††^	NA	−6.7^††^	NA	1.5
**Race and ethnicity**										
American Indian or Alaska Native										
2018	545	22.3	168	31.1	264	39.5	93	15.8	19	—^§§^
2019	546	22.5	160	29.9	266	39.3	96	16.4	24	8.0
2020	588	23.9	176	33.0	285	41.6	103	17.7	23	7.2
2021	692	28.1	196	36.3	365	52.8	96	16.4	35	10.7
2022	650	27.1	166	31.1	343	50.2	111	19.5	29	8.4
2023	577	23.8	133	25.3	312	45.1	105	18.5	27	7.4
2023 vs. 2021 relative rate change, %**	NA	–15.3^††^	NA	–30.3^††^	NA	–14.5^††^	NA	12.4	NA	–30.5
2023 vs. 2018 relative rate change, %**	NA	6.9	NA	–18.6	NA	14.1	NA	16.8	NA	NA
Asian										
2018	1,315	6.7	292	8.5	451	7.2	383	8.2	189	8.0
2019	1,342	6.7	263	7.7	489	7.8	386	8.2	203	8.1
2020	1,302	6.4	253	7.4	466	7.4	365	7.6	218	8.3
2021	1,379	6.8	324	9.4	503	7.9	341	6.9	209	7.8
2022	1,459	6.9	286	8.0	543	8.3	400	7.9	230	8.1
2023	1,407	6.5	297	8.2	518	7.8	369	7.1	223	7.5
2023 vs. 2021 relative rate change, %**	NA	–3.9	NA	−12.4	NA	–1.4	NA	3.1	NA	−3.8
2023 vs. 2018 relative rate change, %**	NA	−3.0	NA	−3.0	NA	8.0	NA	−14.1^††^	NA	−6.5
Black or African American										
2018	3,022	7.3	733	8.2	1,372	11.8	706	7.0	208	4.4
2019	3,115	7.5	749	8.5	1,428	12.1	717	7.1	217	4.4
2020	3,286	7.8	871	9.9	1,523	12.7	659	6.6	223	4.3
2021	3,692	8.7	1,001	11.2	1,758	14.5	693	6.8	234	4.4
2022	3,826	8.9	959	10.7	1,803	14.8	775	7.7	284	5.2
2023	3,911	9.1	945	10.7	1,873	15.2	827	8.2	264	4.7
2023 vs. 2021 relative rate change, %**	NA	4.1	NA	−5.1	NA	4.9	NA	20.3^††^	NA	4.9
2023 vs. 2018 relative rate change, %**	NA	25.2^††^	NA	29.4^††^	NA	29.2^††^	NA	17.4^††^	NA	6.7
Hispanic or Latino										
2018	4,313	7.4	1,105	7.3	1,856	10.3	1,023	8.6	329	7.4
2019	4,331	7.3	1,146	7.5	1,864	10.3	1,042	8.5	274	5.9
2020	4,571	7.5	1,209	7.9	2,077	11.3	944	7.5	335	6.9
2021	4,907	7.9	1,250	7.9	2,291	12.3	1,015	7.8	350	6.9
2022	5,122	8.1	1,251	7.7	2,366	12.6	1,150	8.6	353	6.7
2023	5,281	8.2	1,258	7.7	2,477	12.9	1,150	8.3	394	7.1
2023 vs. 2021 relative rate change, %**	NA	3.4	NA	−2.4	NA	4.6	NA	7.1	NA	1.8
2023 vs. 2018 relative rate change, %**	NA	10.0^††^	NA	5.0	NA	25.2^††^	NA	−3.1	NA	−5.2
Native Hawaiian or Pacific Islander										
2018	73	11.9	19	—	41	21.7	11	—	<10	—
2019	90	14.4	21	16.6	50	25.9	16	—	<10	—
2020	79	12.5	24	18.9	37	18.9	17	—	<10	—
2021	82	12.6	21	16.2	45	22.7	14	—	<10	—
2022	95	14.3	28	21.1	51	25.6	12	—	<10	—
2023	116	17.3	25	18.6	63	31.2	24	16.2	<10	—
2023 vs. 2021 relative rate change, %**	NA	37.4	NA	14.9	NA	37.4	NA	NA	NA	NA
2023 vs. 2018 relative rate change, %**	NA	46.0	NA	NA	NA	43.8	NA	NA	NA	NA
White										
2018	38,415	18.1	4,305	12.9	11,315	23.3	14,489	26.1	8,302	20.7
2019	37,428	17.7	3,976	12.0	11,228	23.0	13,818	25.3	8,401	20.4
2020	35,442	16.9	3,912	12.0	11,076	22.7	12,161	22.6	8,289	19.7
2021	36,681	17.4	4,117	12.4	11,429	23.3	12,371	23.1	8,760	20.9
2022	37,481	17.6	3,647	11.0	11,383	23.3	12,975	24.8	9,473	21.9
2023	37,217	17.6	3,568	10.9	11,404	23.3	12,805	24.9	9,439	21.4
2023 vs. 2021 relative rate change, %**	NA	0.7	NA	−11.9^††^	NA	0.2	NA	7.7^††^	NA	2.7
2023 vs. 2018 relative rate change, %**	NA	−3.1^††^	NA	−14.8^††^	NA	0.1	NA	−4.6^††^	NA	3.6^††^
Multiracial										
2018	514	9.0	162	7.2	215	13.1	107	11.4	29	7.2
2019	527	8.8	166	7.2	231	13.5	110	11.6	20	4.7
2020	599	9.6	189	8.0	275	15.4	109	11.3	26	5.7
2021	631	9.7	203	8.2	294	15.8	99	10.0	35	7.4
2022	682	10.5	190	7.4	317	16.4	151	14.9	24	4.7
2023	628	9.2	183	7.0	284	14.1	123	12.0	37	6.9
2023 vs. 2021 relative rate change, %**	NA	−5.7	NA	−14.2	NA	−10.7	NA	19.9	NA	−6.7
2023 vs. 2018 relative rate change, %**	NA	2.3	NA	−2.8	NA	7.8	NA	4.8	NA	−4.1

**Abbreviation:** NA = not applicable.

* Suicide deaths per 100,000 population. Suicide deaths were identified using *International Classification of Diseases, Tenth Revision* underlying cause-of-death codes U03, X60–X84, and Y87.0.

^†^ Data for Hispanic or Latino (Hispanic) origin should be interpreted with caution; studies comparing Hispanic origin on death certificates and on U.S. Census Bureau surveys have shown inconsistent reporting on Hispanic ethnicity. Potential racial misclassification might lead to underestimates for certain categories, primarily American Indian, Alaska Native, Asian, and Pacific Islander decedents (NationalCenterforHealthStatistics,Series2,no172(8/10/16)). Single-race estimates are presented and might not be comparable to earlier years produced by bridging multiple races to a single race choice. Hispanic ethnicity includes persons of any race. Racial groups exclude persons of Hispanic ethnicity. Persons with unknown ethnicity are excluded from race and ethnicity groups but are included in the overall total.

**^§^** Age-adjusted rates (number of suicide deaths per 100,000 population) were calculated using the direct method and the 2000 U.S. Census Bureau standard population. Ageadjustment-Health,UnitedStates

**^¶^** Includes decedents of unknown ethnicity and children aged <10 years.

** Relative rate change was calculated using the following equations: [(2023 rate – 2018 rate) / 2018 rate] x 100 and [(2023 rate – 2021 rate) / 2021 rate] x 100. Rates were rounded to four decimal places for all calculations and rounded to one decimal place in the table. Data were accessed on CDC WONDER on June 3, 2025.

^††^ Statistically significant at p<0.05 for 2018–2023 and 2021–2023 rate difference based on z-test for at least 100 deaths. If either rate was based on less than 100 deaths, differences in rates were considered significant if CIs based on a gamma distribution did not overlap.

^§§^ Dashes indicate rates are considered unreliable or are suppressed when the rate is calculated with a numerator of <20 or the data meet the criteria for confidentiality constraints (less than 10 deaths).

### Suicide Rates, by Age Group

Between 2018 and 2023, overall U.S. suicide rates declined significantly among persons aged 10–24 (7.0%) and 45–64 years (6.7%) and increased among those aged 25–44 years (5.7%) (Table). Significant increases occurred among Black persons aged 10–24 (29.4%), 25–44 (29.2%), and 45–64 years (17.4%); Hispanic persons aged 25–44 years (25.2); and White persons aged ≥65 years (3.6%). Rates decreased significantly among Asian persons aged 45–64 years (14.1%) and among White persons aged 10–24 (14.8%) and 45–64 years (4.6%).

During 2021–2023, among persons aged 10–24 years, significant declines in rates occurred overall (10.2%) and among White (11.9%) and AI/AN (30.3%) persons. In addition, rates declined significantly among AI/AN persons aged 25–44 years (14.5%). Among persons aged 45–64 years, significant increases in suicide rates were observed overall (6.9%) and among Black (20.3%) and White persons (7.7%).

## Preliminary Conclusions and Actions

Although overall suicide rates remained stable during 2018–2023, rates and changes in rates over time differed by race and ethnicity and age group. Rates increased overall among Black and Hispanic populations and declined overall among White persons and persons aged 10–24 years. Overall suicide rate increases among Black persons are particularly concerning as they continue rate increases described in the previous report (2018 to 2021) (*4*). Conversely, reductions in rates among persons aged 10–24 years are noteworthy given that a previous analysis identified a 62.0% rate increase in this age group during 2007–2021 (NCHSDataBrief,Number471,June2023). During 2021–2023, rates declined among AI/AN persons, overall, after previous increases (*4*); though rates still remain highest in this group. 

There is no single cause of suicide. Rates and rate changes likely reflect the interaction of individual, relationship, community, and societal factors that affect groups differently (*5*). The National Strategy for Suicide Prevention (*1*) calls for a whole-of-society public health approach to prevention. CDC’sComprehensiveSuicidePreventionprogram and its Suicide Prevention Resource for Action (*5*) support populations at increased risk for suicide and prioritize strategies with the best available evidence. Together, community-based approaches, such as strengthening economic supports and increasing connectedness, and health care–based strategies can save lives^†^ (*5*).

## Footnotes

* 45 C.F.R. part 46, 21 C.F.R. part 56; 42 U.S.C. Sect. 241(d); 5 U.S.C. Sect. 552a; 44 U.S.C. Sect. 3501 et seq.

† For persons in crisis, help is available through the U.S. Substance Abuse and Mental Health Services Administration’s 988 Suicide & Crisis Lifeline (https://www.988lifeline.org) or by texting or calling 988.
